# Cancer cachexia: molecular mechanisms and treatment strategies

**DOI:** 10.1186/s13045-023-01454-0

**Published:** 2023-05-22

**Authors:** Tania Setiawan, Ita Novita Sari, Yoseph Toni Wijaya, Nadya Marcelina Julianto, Jabir Aliyu Muhammad, Hyeok Lee, Ji Heon Chae, Hyog Young Kwon

**Affiliations:** 1 Department of Integrated Biomedical Science, Soonchunhyang University, Cheonan-Si, 31151 Republic of Korea; 2 Soonchunhyang Institute of Medi-Bio Science (SIMS), Soonchunhyang University, Cheonan-Si, 31151 Republic of Korea; 3 Institute of Molecular and Cell Biology (IMCB), Agency for Science, Technology and Research (A*STAR), 61 Biopolis Drive, Proteos, Singapore, 138673 Republic of Singapore

**Keywords:** Cancer, Cachexia, Sarcopenia, Treatment, Multi-organ, Muscle wasting

## Abstract

Muscle wasting is a consequence of physiological changes or a pathology characterized by increased catabolic activity that leads to progressive loss of skeletal muscle mass and strength. Numerous diseases, including cancer, organ failure, infection, and aging-associated diseases, are associated with muscle wasting. Cancer cachexia is a multifactorial syndrome characterized by loss of skeletal muscle mass, with or without the loss of fat mass, resulting in functional impairment and reduced quality of life. It is caused by the upregulation of systemic inflammation and catabolic stimuli, leading to inhibition of protein synthesis and enhancement of muscle catabolism. Here, we summarize the complex molecular networks that regulate muscle mass and function. Moreover, we describe complex multi-organ roles in cancer cachexia. Although cachexia is one of the main causes of cancer-related deaths, there are still no approved drugs for cancer cachexia. Thus, we compiled recent ongoing pre-clinical and clinical trials and further discussed potential therapeutic approaches for cancer cachexia.

## Introduction

Skeletal muscle forms 30–40% of the human body mass; hence, it is said to be the most abundant tissue in the human body. For this reason, skeletal muscle is an essential regulator of numerous physiological functions, including body movement. Skeletal muscle is composed of highly organized muscle tissue formed by myofiber bundles through myogenesis [1–3]. The maintenance of skeletal muscle mass depends on homeostasis of the anabolic and catabolic pathways. Anabolism is related to protein synthesis and comprises several important pathways, such as the mammalian target of rapamycin (mTOR), insulin and insulin-like growth factor1 (IGF1)-AKT, and bone morphogenetic protein (BMP)/Smad1/5/8. Catabolic pathways are linked to protein degradation, which includes the ubiquitin (Ub)-proteasome system (UPS), cell autophagy/lysosomal pathway (ALP), and Ca^2+^-activated degradation [2, 4]. The imbalance between these pathways leads to loss of muscle mass and muscle-wasting conditions.

Cachexia is a syndrome characterized by weight and muscle loss (with or without adipose tissue loss) that cannot be entirely reversed by conventional nutritional support. It often occurs as a result of an underlying illness, which can induce various physiological changes including inflammation, loss of appetite or anorexia, low levels of testosterone and other anabolic hormones, and anemia [5, 6]. These underlying illnesses can include cancer, kidney disease, heart failure, neurological disease, chronic obstructive pulmonary disease, and AIDS, among others [7]. Cancer cachexia affects approximately 70% of cancer patients and is responsible for up to 22% of cancer deaths [8]. The pathophysiological mechanism of cachexia is characterized by an imbalance in protein and energy, which is caused by a combination of reduced food intake and abnormal metabolism. This condition also affects the ability of muscles to regenerate [8–12]. Cancer cachexia has three clinically relevance stages: precachexia, cachexia, and refractory cachexia. In precachexia, early clinical and metabolic signs, such as anorexia and impaired glucose tolerance, lead to significant weight loss of 5% or less. Cachexia is diagnosed in patients with weight reduction of more than 5%, or a weight loss of over 2% in individuals who are already depleted based on their current body weight and height (with a body mass index [BMI] below 20 kg/m^2^) or skeletal muscle mass. The refractory stage is characterized by advanced cancer that is unresponsive to treatment [13].

Cancer cachexia has been identified as a negative outcome of cancer, leading to reduced physical function, tolerance to anticancer therapy, and survival rates [8, 12, 14]. Despite this, specific therapies for cancer cachexia are limited. Here, we describe skeletal muscle atrophy and the molecular mechanisms that affect muscle wasting in cancer cachexia. Moreover, potential treatment options and up-to-date clinical trials of cancer cachexia are discussed. Therefore, this review will concentrate on the causes of muscle wasting, the underlying molecular mechanisms, and potential treatment options for cancer cachexia.

## Cancer cachexia as a multi-organ syndrome

### Brain and food intake

**Fig. 1:**
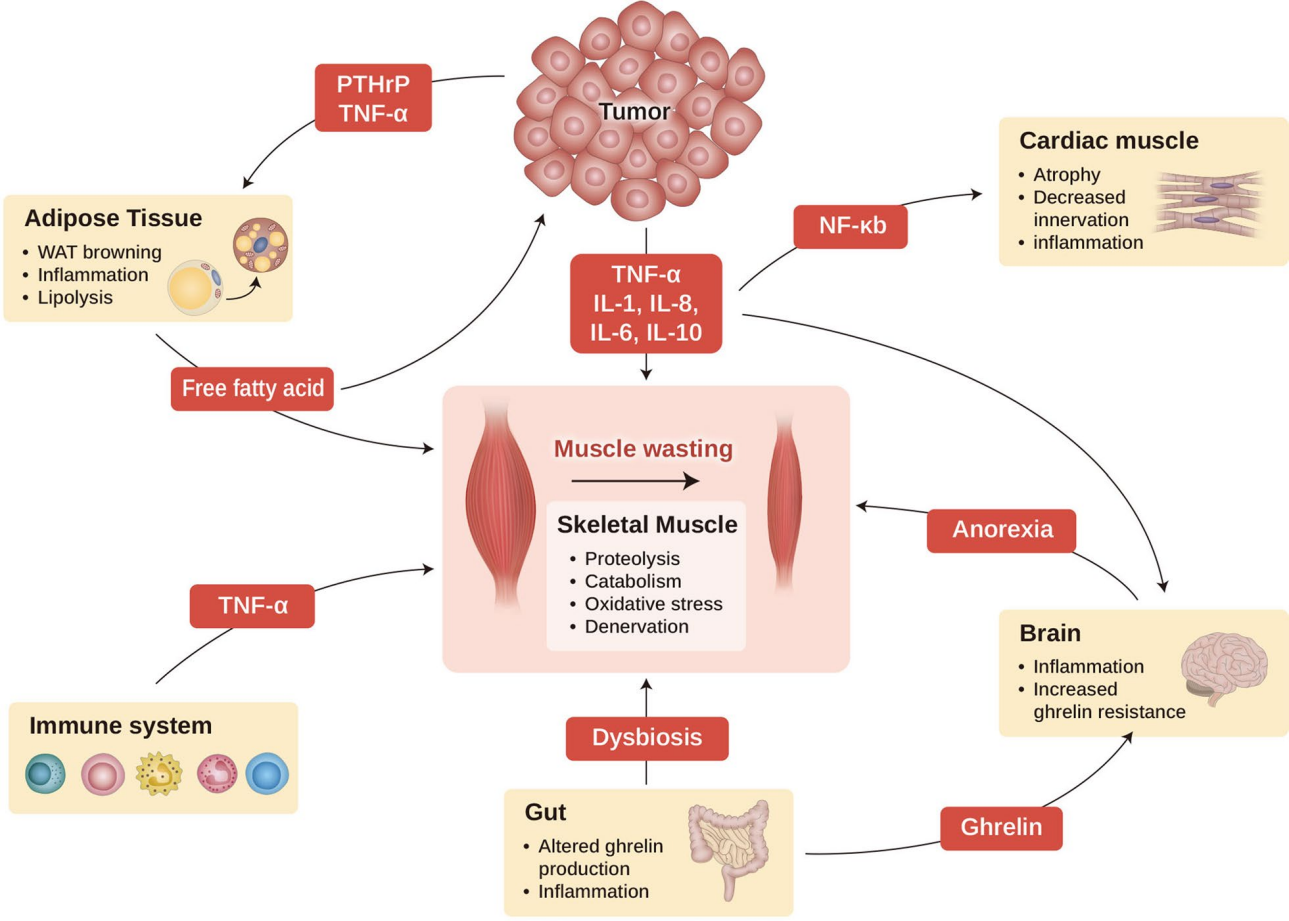
Cancer cachexia as multi-organ syndrome. This scheme shows the interaction of major organs that are associated with and commonly affected by cachexia. Cancer cachexia that happens in the muscle (center) is dependent on the alterations in other organs, such as adipose tissue, brain, gut, cardiac muscle, and immune cells. Cachexia-inducing tumors secrete many factors, such as cytokines, PTHrP, and other mediators, to induce muscle wasting directly, as well as affecting other organs such as brain, cardiac muscle, gut, and adipocyte tissue, which aggravates cachexia syndrome. WAT, white adipocyte tissue; PTHrP, parathyroid hormone-related protein; TNF-α, tumor necrosis factor-α; IL-1, interleukin-1; IL-6, interleukin 6; IL-8, interleukin-8; IL-10, interleukin 10; and NF-kB, nuclear factor kappa-light-chain-enhancer of activated B cells

Cachexia is a condition that is characterized by malnutrition, weight loss, depletion of muscle mass, anorexia, fat metabolic disorder, inflammation, gut dysbiosis, and frailty (Fig. 1) [13, 15]. The loss of weight and muscle mass can be attributed to several factors such as decreased food intake, anorexia, insulin resistance, and low levels of anabolic hormones [6]. The involvement of the neuroendocrine system in cachexia and the role of the hypothalamus, pituitary gland, and adrenal gland in controlling appetite have recently gained attention in cancer cachexia study [16–21]. The hypothalamus is the key regulator of energy homeostasis, especially in fine-tuning the energy balance by delivering signals to coordinate food intake and vice versa, suppressing energy expenditure [22]. Hypothalamic neuropeptide Y (NPY) and agouti gene-related protein (AgRP) neurons induce appetite, whereas proopiomelanocortin (POMC) and cocaine- and amphetamine-regulated transcript (CART) neurons suppress it [16, 17, 19–21, 23].

The decreased activity of NPY/AgRP neurons appears synergistically with hyperstimulation of POMC neuronal cells [24]. In the brain, the nucleus of the solitary tract and the melanocortin system have also been implicated in the control of appetite and neuroendocrine-axis-mediated cancer cachexia [18, 23, 25]. Another neuronal circuit that has been found to be dysregulated in cancer cachexia is the hypothalamic serotonergic and dopaminergic systems [26, 27]. Anorexia in cancer cachexia is also strongly associated with chronic inflammation, which promotes the expression of pro-inflammatory cytokines in the hypothalamus, leading to the inactivation of NPY/AgRP neurons and activation of POMC/CART neurons [24, 28, 29]. Excessive cytokine production in cancer also increases the expression of corticotrophin-releasing factor, a potent anorectic agent, which, in concert with prostaglandins, suppresses the production of NPY [30, 31]. Cytokines can also cause a delay in gastric emptying, lower albumin concentration, and enhance lipolysis [32]. Aside from pro-inflammatory cytokines, parathyroid hormone-related protein (PTHrP) has also been implicated in cachexia [33, 34]. PTHrP decreases food intake and promotes muscle wasting by activating hypothalamic urocortins 2/3 through vagal afferent pathways and inhibiting gastric emptying [33]. It has been shown that PTHrP neutralization is sufficient to suppress the β-adrenergic timbre, which attenuates energy expenditure and muscle loss in anorectic mice [34]. Anorexia can be enhanced by physical symptoms, such as pain, fever, abdominal pain, diarrhea, respiratory problems, and several psychiatric symptoms [28, 29, 35].

Ghrelin, responsible for controlling appetite, is strongly secreted in cachexia [36]. Its secretion the stomach can be induced by a compensatory mechanism that buffers cachexia [37]. Ghrelin performs various activities, such as increasing fat [38], preventing muscle atrophy [39], and decreasing muscle breakdown [40]. The GI tract or ghrelin secreted by the stomach can be induced by a compensatory mechanism that buffers cachexia [37]. Ghrelin is a 28-amino acid neuropeptide hormone released from the stomach in response to fasting that stimulates food intake [41]. Ghrelin is responsible for controlling appetite and strongly secreted in cachexia [36]. Ghrelin performs various activities, such as increasing fat [38], decreasing [40] and preventing muscle atrophy [39]. Ghrelin receptors are growth hormone receptors and are expressed in the hypothalamus and pituitary gland. They are known to mediate growth hormone release and improve appetite, but much research is needed on their mechanism of action [42, 43]. According to many studies, ghrelin is known to suppress inflammation by releasing the anti-inflammatory cytokine IL-10 [44], which reduced the levels of interleukin-1β (IL-1β), IL-6, and TNF-a [45]. In addition, according to Chen’s report, ghrelin prevented the reduction of muscle mass by inhibiting the NF-kB-mediated ubiquitin–proteasome pathway [46]. The administration of ghrelin to mice attenuated dexamethasone-induced muscle atrophy by inhibiting the expression of Atrogin-1 and MuRF1 through PI3Kβ-, mTORC2-, and p38-mediated pathways [39]. Anamorelin is a ghrelin receptor agonist that is used in cancer treatment. It promotes ghrelin secretion through ghrelin receptor activation and increases appetite, resulting in increased weight and muscle mass [47]. In early clinical trials, anamorelin improved skeletal muscle mass and appetite [48, 49]. However, elevated ghrelin levels are reported in patients with cancer cachexia [50]. In addition, a study conducted on rats with patient-derived cancer showed that despite the elevation of ghrelin, appetite and energy storage failed to increase due to ghrelin resistance [51]. Thus, the effect of ghrelin in patients with cancer cachexia remains a matter of debate.

### Adipose tissue

Cancer cachexia is also associated with adipose tissue (AT) wasting, leading to profound weight loss and frailty [52]. White adipose tissue (WAT) and brown adipose tissue (BAT) are complex tissues that are important for maintaining metabolic homeostasis. In patients with cancer cachexia, metabolic and histo-morphological alterations in AT lead to wasting, which promotes muscle loss [53–56]. WAT browning, a condition in which WAT acquires BAT characteristics, leads to an actively promoting systemic and local catabolic state that ultimately induces lipolysis and adipokine secretion. In rodent models and patients with cancer cachexia, metabolic changes to white adipocyte browning lead to the activation of BAT and UCP1, thereby promoting thermogenesis, which increases energy expenditure and causes an energy imbalance. More recently, it has been reported that Lewis lung carcinoma cells (LLC)-derived extracellular vehicles (LLC-EVs) deliver PTHrP to interact with the parathyroid hormone receptor (PTHR). This interaction induces phosphorylation of protein kinase A (PKA), an enzyme that induces lipolysis and activates UCP1, thereby increasing WAT browning and thermogenesis [57, 58]. PTHrP is also secreted by tumor cells and inhibits adipocyte differentiation through peroxisome proliferator-activated receptor gamma (PPARγ) activity inhibition via a mitogen-activated protein kinase (MAPK)-dependent pathway [34, 52]. Moreover, patients with cancer cachexia often develop AT fibrosis, which indicates uncontrolled remodeling of the extracellular matrix. This may cause alterations in AT metabolism, ultimately resulting in the induction of TGF-β and SMAD expression in subcutaneous adipose tissue (SAT) [54]. Han et al. reported that patients with cancer cachexia with lower SAT values had worse prognosis compared to patients with higher SAT values [59]. Inflammation also induces peripheral and systemic changes. Inflammation in the early stages of cancer cachexia induces WAT lipolysis; however, in the late stages of cancer cachexia, it is correlated with the induction of WAT browning. Moreover, TNF-α levels are elevated in the AT of tumor-bearing rats, which contributes to muscle wasting and reduced AT mass. TNF-α is known to induce inflammation and reduce the expression of GLUT4, resulting in decreased glucose uptake by skeletal muscles from the bloodstream [60, 61].

#### Immune system

The role of the immune system in cancer cachexia cannot be neglected, as one of the biggest drivers of cancer cachexia and muscle wasting is strongly associated with the release of pro-inflammatory factors. The release of TNF-α by immune and non-immune cells has been linked directly to muscle wasting by activating the UPS [62, 63]. TNF-α exerts its catabolic function in a pleiotropic manner by stimulating muscle protein degradation through the activation of the E3 ligase pathway [44]. Moreover, TWEAK, a TNF-related weak inducer of apoptosis, has recently been reported as a cytokine that induces skeletal and cardiac muscle atrophy by activating the UPS [64]. In addition, increased levels of TNF-α, IL-1, IL-8, and IL-10 in patients with cancer cachexia result in increased energy expenditure, loss of appetite, and muscle atrophy [65]. IL-1 increases the expression of atrogin-1/muscle atrophy F-box (MAFbx) and muscle RING-finger 1 (MuRF1), two important E3 ligases, which mark myofibrillar protein of C2C12 myotubes for proteasomal degradation [66]. Meanwhile, IL-8 secretion in pancreatic cancer was shown to induce muscle atrophy via the CXCR2-ERK1/2 axis [67].

In addition to various cytokines, innate immune cells, such as macrophages and myeloid-derived suppressor cells (MDSCs), as well as adaptive immune cells, particularly T cells, are thought to play a role in cancer cachexia. M2 macrophage infiltration escalates myo-degradation in pancreatic cancer models through activation of the STAT3 signaling pathway. In contrast, M2 depletion results in reduced systemic inflammation and muscle atrophy [68]. These findings indicate a negative role of M2 macrophage in cancer cachexia. However, the exact mechanism by which MDSC expansion could result in cancer cachexia remains unclear. However, MDSCs expansion observed in the 4T1 mouse model significantly increases oxygen consumption, which is one of the characteristics of cachexia. The mouse model also showed a significantly higher loss of total adipose tissue compared to cells without MDSC expansion and the non-tumor model [69]. These findings suggest that MDSCs play a potential role in driving cancer cachexia. In contrast, there was a positive correlation between T cell infiltration and protection against cachexia. CD4^+^ Treg cells are known to protect muscle fibers from atrophy [70, 71]. Meanwhile, CD8^+^ T cells were shown to be inversely associated with several signaling pathways regulating the maintenance of muscle mass, such as ubiquitin–proteasome (TRIM63, UBE2B, UBE2L3, UBA52, MUL1, FBXO32, UBB, UBC, USP4, and DNAJC11), catabolic signaling (ACVR2B and ACVR1B receptors), apoptosis (CASP8 and SIVA1), and autophagy (ATG13) [72]. In general, immune cells can function as either pro-cachectic or anti-cachectic agents. The development of cancer cachexia is dependent on the patients’ immune system, and thus, the interplay between inflammatory cytokines, immune cells, and pro-cachectic factors require further exploration.

### Gut

The gastrointestinal tract of mammals consists of trillions of microbes, which are now considered full components of the body. The gut microbiota plays an important role in nutrient utilization, maturation of the immune system, resistance against infections, and host metabolism. Recent studies have suggested that the composition of the gut microbiota is affected by tumor cells [73]. Although cancer cachexia is particularly related to gastrointestinal (GI) tumors, patients with upper GI tract cancer have a higher prevalence of cachexia than other patients [74]. Patients with cancer who receive radiation or chemotherapy often suffer from intestinal wall dysfunction due to leakage of the intestinal epithelial barriers [44]. Changes in intestinal composition and components of the mucosal barrier alter the usual equilibrium of the microbiota, both in the abundance and diversity of bacteria, which results in intestinal dysbiosis in which the dominance of a particular taxon is frequent [75]. In patients with cancer, a barrier with increased permeability due to decreased expression of Zonula occludens-1 (ZO1), a tight junction-associated protein, and occludin, an NADH oxidizing enzyme, causes bacteria and bacterial cell wall components (endotoxins or lipid polysaccharides) to easily enter the circulatory system and cause inflammation [76].

High-permeability barriers can also cause diarrhea, which can lead to energy imbalances and absorption disorders [44]. A study using a colon cancer mouse model (transgenic APC^±^ strain) reported that the barriers were destroyed due to tumor growth, resulting in increased systemic inflammation and endotoxemia, which further caused profound inhibition of gastrointestinal motility [77]. This model is used as mice exhibit excessive weight loss as the tumor burden progresses. Another animal model, where C26 colon adenocarcinoma cells were ectopic transplanted, was used to study the interaction between cachexia, gut barrier dysfunction, and microbiome. The study revealed significant alterations in the intestinal homeostasis accompanied by changes in microbial composition and increased gut permeability. These changes were also associated with an increase in pro-inflammatory bacterial translocation, which is relevant to clinical data from cachetic patients with lung and colon cancer [73].

### Cardiac muscle

Cancer cachexia plays a role in cardiac muscle wasting, which eventually leads to heart dysfunction and remodeling [78]. A study showed that mice with cancer cachexia had smaller hearts with reduced wall thickness compared to those in healthy controls [79]. Factors secreted by muscles, such as myostatin and GDF15, or factors secreted by cancer-associated immune cells initiate a series of processes that cause cancer cachexia [15]. Recent studies have shown that during tumor growth, the nitrogen balance is managed by tumors, which results in metabolic alterations, such as cardiomyocyte atrophy and aberrant lipid metabolism [15]. Cachexia-induced metabolic changes, such as autophagy, increased energy expenditure, sequestration of nutrients by tumors, and proteolysis, can collectively contribute to reduced oxidative capacity of the heart muscle, impaired mitochondrial homeostasis, and muscle atrophy. Consequently, these metabolic alterations can trigger a cascade of events that ultimately lead to heart failure due to cardiac muscle damage during cachexia [80, 81].

The combined action of oxidative stress and cytokines activates NF-κB/TNFα signaling which decreases the activity of PGC-1α, a transcription factor that promotes oxidative capacity [82–84]. The release of calcium by the mitochondria stimulates the secretion of cytochrome C, which can lead to disrupted mitochondrial homeostasis [81]. Furthermore, the release of cytochrome C activates caspases by forming an apoptosis-initiating complex with apoptotic protease activating factor-1 (Apaf-1), dATP, and pro-caspase-9. The activation of pro-caspases-9 leads to the activation of pro-caspase-3, which initiates a cascade of caspases [81, 85–87]. These alterations cause heart failure in patients with cancer cachexia. In addition, some studies have shown that cardiac remodeling induced by cachexia is related to the deterioration of nerve function in an LLC model. This may be due to a decrease in the expression of nerve growth factors in tumor-bearing mice [88]. The remodeled heart and cancerous cells show close exchange through the secretion of multiple factors that trigger cachexia [89]. However, the specific mechanisms underlying cardiac impairment in cancer cachexia are still unclear; hence, further studies, including cardiac-gut and cardio-cerebral inter-organ interactions, are necessary.

### Skeletal muscle

Skeletal muscle wasting is a significant feature of cancer cachexia, a severe and complex condition [78, 90]. Patients with cancer cachexia experience a notable reduction in their actual body weight and body mass index, mainly due to a cancer-related weight loss of around 22.3% on average, with a monthly weight loss rate of 3.45 ± 0.79% [90]. In addition, studies have shown that cachectic cancer patients have a 10–33% reduction in quadriceps muscle area and 4–13% reduction in skeletal muscle index compared to healthy individuals [91, 92]. Consequently, the quality of muscles is compromised in cancer cachexia patients, significantly impacting their quality of life [91]. Furthermore, gene expression pattern analysis of muscles affected by cachexia conditions revealed a set of genes called atrogenes, including several genes responsible for cellular degradation systems such as the ubiquitin–proteasome and autophagy-lysosome systems, which play a critical role in muscle atrophy [11].

Three main signaling pathways have been identified to contribute to skeletal muscle degradation: UPS, ALP, and Ca^2+^ activated degradation pathways [93]. The UPS is an active protein degradation pathway that degrades ubiquitinated proteins in cells [94, 95]. It is the most significant signaling pathway involved in skeletal muscle protein degradation [94, 95]. The second common pathway is autophagy, which is a highly conserved process in eukaryotes that occurs in the cytoplasm and transports abnormal or excess organelles to lysosomes for degradation [96, 97]. A highly activated UPS pathway seems to play an essential role in inducing muscle wasting in cancer cachexia [93, 98, 99]. Several studies using various preclinical cancer cachexia models and patients have been conducted to provide a better understanding of muscle wasting induced by cachexia [100]. A study using muscle biopsies from patients with cancer cachexia showed that the expression of ubiquitin mRNA and 20S proteasome subunits was upregulated [101]. In addition, muscle proteasome activity was increased in patients with cachexia compared to controls [102]. The UPS consists of ubiquitin, ubiquitin-activating enzyme (E1), ubiquitin-conjugating enzyme (E2), ubiquitin ligase (E3), protease, and its substrate [103]. E3 ligase targets proteins for degradation by identifying and binding to specific target protein sequences [103]. In skeletal muscle, E3 ligase muscle-specific RING finger protein-1 (MuRF1) and muscular atrophy fbox-1 protein (MAFbx/Atrogin-1) are two key ligases that identify muscle proteins to be degraded by the UPS [104]. MuRF1 and Atrogin-1 are regulated by a variety of signaling pathways, including NF-κB, IL-6, and the p38 MAPK pathway [66, 105–107].

Autophagy plays an essential role in the selective elimination of damaged organelles and degradation of misfolded proteins [108]. mTOR and AMPK are key regulators of autophagy that act sensors to maintain energy balance within cells [109, 110]. There is growing interest in the role of autophagy in mediating skeletal muscle wasting and cachexia progression [93, 98]. Accumulating evidence suggests that autophagy is highly upregulated during cancer cachexia [111, 112]. Elevated levels of autophagy mediators, such as BNIP3A messenger RNA and LC3B protein, have been observed in a small cohort of patients with lung cancer [112]. Moreover, autophagy was also reported to be the main driver of skeletal muscle proteolysis in esophageal and gastrointestinal cancers [111, 112].

A basal level of autophagy is important for maintaining healthy cell function [109, 110]. A study using a C26 cancer cachexia mouse model showed that beclin knockdown failed to prevent muscle atrophy in tumor-bearing mice and that tumor protein p53-inducible nuclear protein 2 (TP53IN P2)-mediated autophagy aggravated muscle loss [113]. Furthermore, specific deletion of the autophagy gene Atg7 causes severe muscle atrophy, reduced force production, and abnormal mitochondrial biogenesis [109, 110]. While the balance of autophagy is essential for normal cell functioning, autophagy-associated markers can be upregulated in skeletal muscle cells under catabolic conditions [114–116]. FOXO3 was reported to be the main transcription factor that induces autophagy and regulates the expression of autophagy genes, including LC3 and Bnip3 [99]. Activated FOXO3 stimulates ALP by reducing IGF1/PI3K/AKT signaling pathway activity via mTOR and transcriptional dependent mechanisms [99]. In another study, oxidative stress was reported to be related to the induction of ATG7 expression in the ALP pathway, which is also associated with the p38 MAPK pathway [107].

Calcium plays an essential role in regulating the binding of calpastatin to calpain, resulting in the inhibition of calpain activity [117]. Calpains belong to a large family of calcium-dependent cysteine proteases that cleave the myofibrillar proteins to disrupt sarcomeres [118]. To date, little is known about the relevance of Ca2 + -activated degradation pathway in cancer cachexia. Nonetheless, a previous study has shown that calcium is involved in the regulation of glucocorticoid-induced muscle proteolysis [119]. Treatment of L6 myotubes with BAPTA, a calcium chelator, or with a calmodulin kinase II inhibitor (KN-62) significantly ameliorated the increase in dexamethasone-induced protein degradation [119]. Moreover, the mRNA levels and activity of calpain were found to be increased in the skeletal muscle of old rats compared to young rats, which was further inhibited by the administration of calpastatin [120]. An in vitro cachexia model using liver cancer cells and a C26 colon cancer study demonstrated that Calpain-1 was highly upregulated in these cells, which was opposite to the calpastatin level, and the Ca2 + -dependent proteolytic pathway was highly activated in the C26 cachexia rat model [121]. Similarly, activation of Ca2 + -dependent proteolysis has also been reported in the skeletal muscle and heart in cancer cachexia [117]. Another study also reported that proteolysis-inducing factor (PIF) prompts muscle loss in cachexia by its high-affinity membrane-bound receptor [122]. In skeletal muscles, PIF binding to its receptor induces a high accumulation of Ca2 + leading to the activation of a Ca2 + -dependent degradation system, which results in an increase in skeletal muscle protein degradation [122].

### Alterations of anabolic pathways in cancer cachexia

**Fig. 2:**
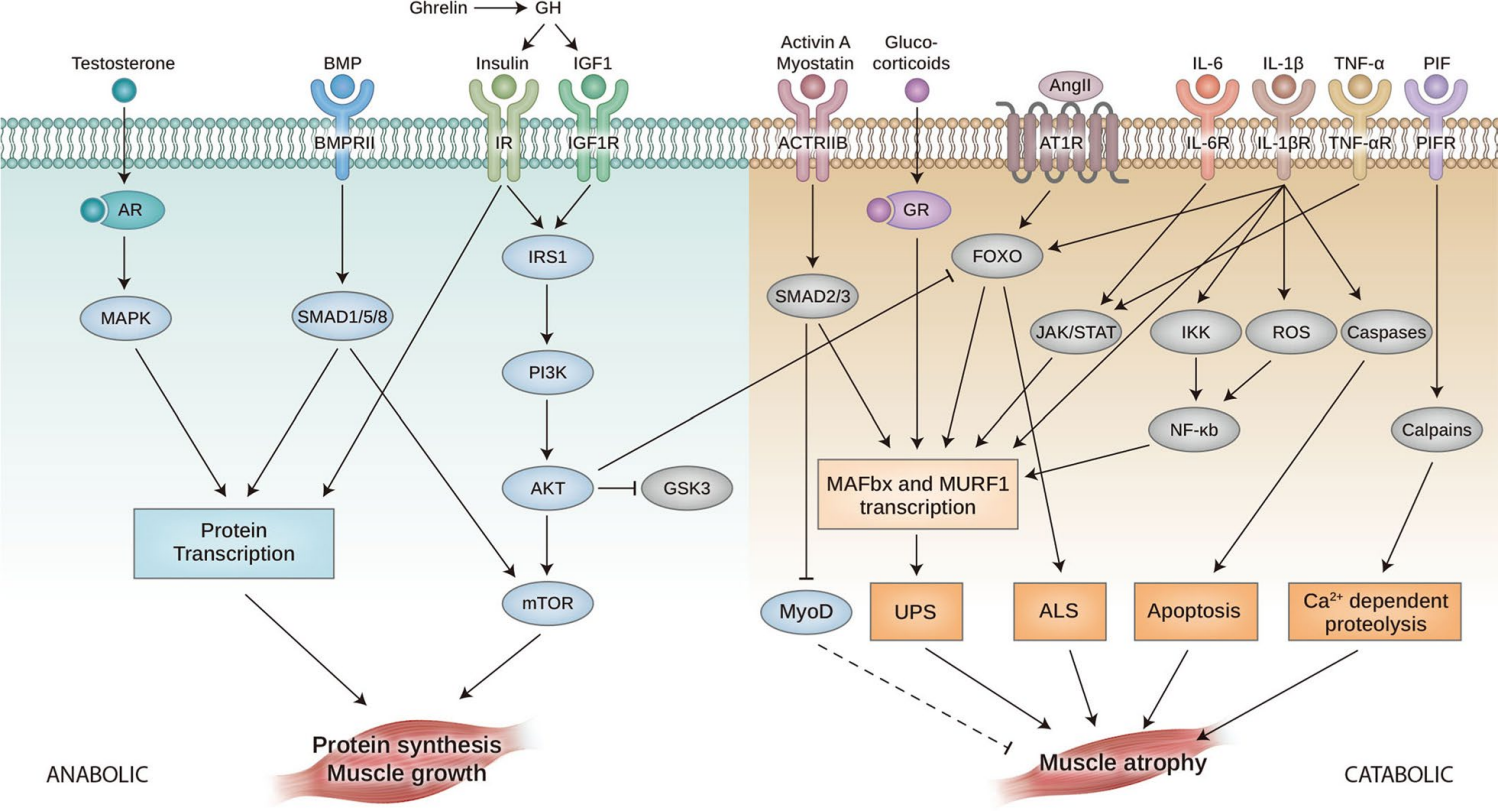
Muscle anabolic and catabolic signalings involved in muscle growth and wasting. Growth factors and nutrients activate PI3K-AKT-mTOR pathway, resulting in an increase in muscle protein synthesis. Furthermore, MAPK and SMAD 1/5/8 activation also induces protein transcription, leading to muscle growth. Conversely, in cachexia conditions, inflammatory cytokines from tumors and immune cells induce activation of transcription factor NF-kB, leading to UPS and ALS activation, which leads to muscle wasting. Furthermore, activin and myostatin bind to the ActRIIB, which phosphorylates SMAD2/3, activating UPS. Glucocorticoid and AngII also activate UPS and ALS pathway, respectively, and lead to muscle wasting. GH, growth hormone; IGF1R, IGF1 receptor; IR, Insulin receptor; BMP, bone morphogenetic protein; BMPRII, BMP receptor II; AR, androgen receptor; ActRIIb, activin type II receptor; AngII, Angiotensin II; AT1R, type 1 angiotensin II receptors; IL-6R, Interleukin 6 receptor; IL1bR, IL1b receptor; TNFaR, TNF receptor; PIF, proteolysis-inducing factor; PIFR, proteolysis-inducing factor receptor; GR, glucocorticoid receptor; ROS, Reactive oxygen species; UPS, ubiquitin (Ub)-proteasome system; and ALS, autophagy-lysosome system. The dashed lines indicate inhibited pathways

As skeletal muscle is mainly composed of proteins, the regulation of homeostasis in protein synthesis and degradation is essential. There are two pathways regulating skeletal muscle homeostasis: the anabolic and catabolic pathways (Fig. 2). An imbalance between anabolic and catabolic pathways in muscle metabolism caused by chronic diseases, such as cancer, leads to muscle wasting, as summarized in Table 1. The anabolic pathway stimulates muscle growth by increasing protein synthesis, resulting in the accumulation of proteins and organelles in the cytoplasm. Several signaling pathways that promote muscle growth have been described.

**Table 1:** Major component and deregulated signaling pathways in cancer cachexia

Pathway	Signaling	Regulator	Signaling implicated	Function	Expression	References
Anabolic	mTOR	mTORC1	S6 kinase phosphorylation	Regulates protein synthesis	Decreased	[11, 123, 124]
			FGF21 induction and mitochondrial biogenesis	Regulates muscle growth and positively associated with muscle mass		
		mTORC2	Phosphorylates PKB/Akt on Ser473	Regulates glucose and lipid homeostasis		[11, 124]
	Insulin and IGF1-AKT	IGF1	mTOR activator	Regulates protein synthesis and degradation, cellular proliferation, glucose uptake, and energy production		[11, 132]
			P13K/AKT activation			[150]
			Suppression of MuRF1 and Atrogin-1			[150]
		AKT	Inhibits the NF-kB pathway and the FoxO protein	Regulates muscle growth		[137]
		Plakoglobin	Activation of PI3K-AKT-FoxO signaling	Controls muscle growth and metabolism		[151]
	BMP/Smad1/5/8	BMP	Smad1/5/8 phosphorylation	Positive regulator of muscle mass growth		[154, 155]
		BMP7	Activation of Smad1/5/8 signaling	Regulates protein synthesis		[157]
Catabolic	Ubiquitin–proteasome system	Myostatin	Represses the Akt/mTOR pathway; activates SMAD2 and SMAD3 transcription; upregulates MuRF1, MAFbx/Atrogin1, and FOXO expression	Positive regulator of protein breakdown	Increased	[173, 174, 177, 180]
		Activin A				[173, 177, 180]
			Induces BMP inhibitor Noggin expression	BMP activity inhibitor in muscle fibers and motor nerves		[156]
		Smad2/3	Regulates myostatin and activin A expression	Negative regulators of muscle growth		[154, 155]
	NF-κB	NF-κB	IGF1 inhibitor	Regulator of muscle cell death and specific transcriptional regulation		[189, 190]
			Suppresses MyoD expression			
			Increases MuRF1 expression	Positive regulators of proteolysis of skeletal muscle proteins		[306]
			iNOS/NO pathway regulator	Negative regulator of skeletal muscle growth		[191, 307]
		NIK	increases the levels of atrophy markers			[306]
	Inflammatory cytokines	TNF-α	NF-κB activation; involved in the ubiquitin conjugation and proteasomal degradation of iKb	Positive regulators of proteolysis of skeletal muscle proteins		[189, 190, 197]
			induces the expression of ubiquitin genes in the UPS and activates the p38 MAPK pathway	Stimulates both reactive oxygen production and general activity of the ubiquitin-conjugating pathway		[164]
	IL-6-JAK-STAT3	STAT3	Stimulates mitochondrial respiration	Promotes myogenic lineage progression in muscle stem cells		[198–200]
			Increase expression of myostatin, MAFbx, and MURF1	Regulates skeletal muscle mass in myofibers		[207, 208]
			Activation of the IκB kinase (IKK)/NF-κB signaling pathway	Positive regulators of apoptosis		[307]
			Stimulates C/EBPδ expression and activity			[207, 208]
		IL-6	Induces STAT3 phosphorylation	Positive regulators skeletal muscle proteolysis		[162, 204, 205]
			Activation of IL3			[[206]
			AMPK activation and suppression of mTORC1 activation			[133]
			Induces BMP inhibitor Noggin expression	Negative regulator of BMP activity in muscle fibers and motor nerves		[156]
	Cell autophagy/lysosomal pathway (ALP)	BNIP3A	Autophagy mediators	Selective elimination of damaged organelles and degradation of misfolded proteins		[108, 112]
		LC3B		Driver of skeletal muscle proteolysis		[111, 112]
		FOXO3	Main transcription factor that induces autophagy	Regulates the expression of autophagy genes		[99﻿]
			Reduces IGF1/PI3K/AKT signaling pathway activity via mTOR and transcriptional dependent mechanisms	Positive regulator of ALP		
		ATG7	Regulator of p38 MAPK pathway			[107]
	Ca2 + -activated degradation	Calcium	Regulates the binding of calpastatin to calpain	Calpain activity inhibitor		[117]
			Regulation of glucocorticoid	Driver of skeletal muscle proteolysis		[119]
		Calpains	Cleaves myofibrillar proteins	Disrupt sarcomeres		[118, 120, 171]
		Proteolysis-inducing factor (PIF)	Induces a high accumulation of Ca^2+^	Positive regulator Ca^2+^-dependent degradation system		[122]

### mTOR

The mechanistic target of rapamycin (mTOR) is a central growth factor as well as a nutrient and stress regulator [123, 124]. mTOR contains mTOR complex 1 (mTORC1) and mTOR complex 2 (mTORC2), of which mTORC1 regulates anabolic processes, such as protein synthesis, and ribosomal and mitochondrial biogenesis, and mTORC2 regulates glucose and lipid homeostasis [11, 124]. mTORC1 activation induces phosphorylation of S6 kinase (S6K) and eukaryotic translation initiation factor 4E–binding protein (4E-BP), which leads to protein synthesis. Therefore, mTORC1 regulates muscle growth and is positively associated with muscle mass. mTORC1 regulates metabolic homeostasis by activating 4E-BP1, which in turn induces FGF21 and increases the translation of peroxisome proliferator-activated receptor γ coactivator-1α (PGC-1α). PGC-1α enhances mitochondrial biogenesis by increasing oxygen consumption and oxidative function [123–126].

As mTORC1 regulates muscle homeostasis and controls muscle autophagy [11, 127], aberrant expression of mTORC1 during muscle growth, whether through inhibition or upregulation, can lead to muscle atrophy. In mTOR-deficient mice, the absence of mTOR results in reduced postnatal growth due to a reduction in fast fibers [11]. Depletion of Raptor in muscle progenitors is prenatally lethal and affects muscle development [128]. Thus, in the growth stage, inactivation of mTORC1 results in lethal myopathy [129]. However, the dispensability of mTORC1 in adult muscle maintenance has been reported by Ham et al. [130]. They found that raptor depletion specifically in fully grown muscle using inducible-skeletal muscle-specific deletion of raptor in young adult mouse for 21 days did not affect muscle mass and function; instead, it affected the muscle translation machinery for protein synthesis. The results indicate that, in mature adult animals, a significant portion of basal protein synthesis occurs independently of mTORC1, and muscle maintenance in sedentary mice is largely independent of mTORC1 activity [130]. Similarly, rapamycin treatment in young adult rats (10–12 weeks old) did not impact muscle mass. However, initiating rapamycin treatment in 2-week-old rat pups for 14 days caused a 40% reduction in hindlimb muscle mass [131]. Therefore, although mTORC1 signaling is required for muscle development in young adult animals, it appears to be dispensable for maintenance in mature adult animals [11, 127–129].

Activation of the mTOR pathway through insulin-like growth factor-1 (IGF1) is also reduced in tumor-bearing mice with cancer cachexia [132]. It has been reported that upregulation of IL-6 in cancer cachexia leads to suppression of mTORC1 activation through AMP-activated protein kinase (AMPK) activation [133]. During the initiation of cancer cachexia, muscle protein synthesis is reduced. This is associated with IGF1/mTOR signaling repression. Muscle mTOR activation is reduced after at least a 12% loss of body weight and further decreases during cachexia progression. During the later stage of cachexia, muscle AMPK is activated, which leads to mTOR signaling repression. [134]. Moreover, a clinical study using a long-term mTOR inhibitor showed the induction of muscle mass loss by significantly decreasing skeletal muscle area and lean body mass [135]. Chen et al. reported that cachexia models induced by CT-26 and LLC tumors show a reduction in mTOR and mTOR phosphorylation [136]. Similarly, another group also showed impairment of mTORC1 during cancer cachexia using the LLC1 and C26 colon cancer model, which is associated with a 57% reduction in protein synthesis rates [137]. Consistently, mTOR inhibition leads to a decrease in body weight, food intake, and fat mass [136].

Chronic activation of mTORC1, on the other hand, has been reported to induce the molecular signature of sarcopenia in recent studies. In both aging mice and humans, mTORC1 signaling is hyperactivated and colocalized with fiber damage, leading to muscle wasting [123, 138]. The induction of oxidative damage and apoptosis-related genes by mTORC1 activation is only apparent in aged mice, and significant degenerative morphology is only noticeable after the age of 30 months, causing progressive oxidative stress, fiber damage, and fiber loss [123]. Although the upstream regulator of mTORC1 elevation in aging is unknown, multiple factors, such as altered proteostasis, inflammation, and NMJ instability, are likely to play a role and may be regulated by changes in mTORC1 activity [123, 139]. In addition, mTORC1 activity contributes to age-related muscle atrophy and GDF signaling, and increased GDF15 leads to the phosphorylation of STAT3 [123, 138]. Thus, inhibition of mTORC1 can alleviate muscle wasting in aging skeletal muscles, and rapamycin treatment can improve muscle function in muscular dystrophies [128]. In contrast, overexpression of mTORC1 through AKT activation leads to muscle hypertrophy [123]. However, identifying the optimal dose and dosing strategy for mTORC1 inhibition will be crucial for its viability as an anti-aging therapy in future studies. Additionally, given that mTORC1 activation should be carefully balanced to maintain homeostasis of skeletal muscle mass, particularly in older cancer patients, caution should be exercised when considering its use as an anti-aging therapy.

### Insulin and IGF1-AKT

**Fig. 3:**
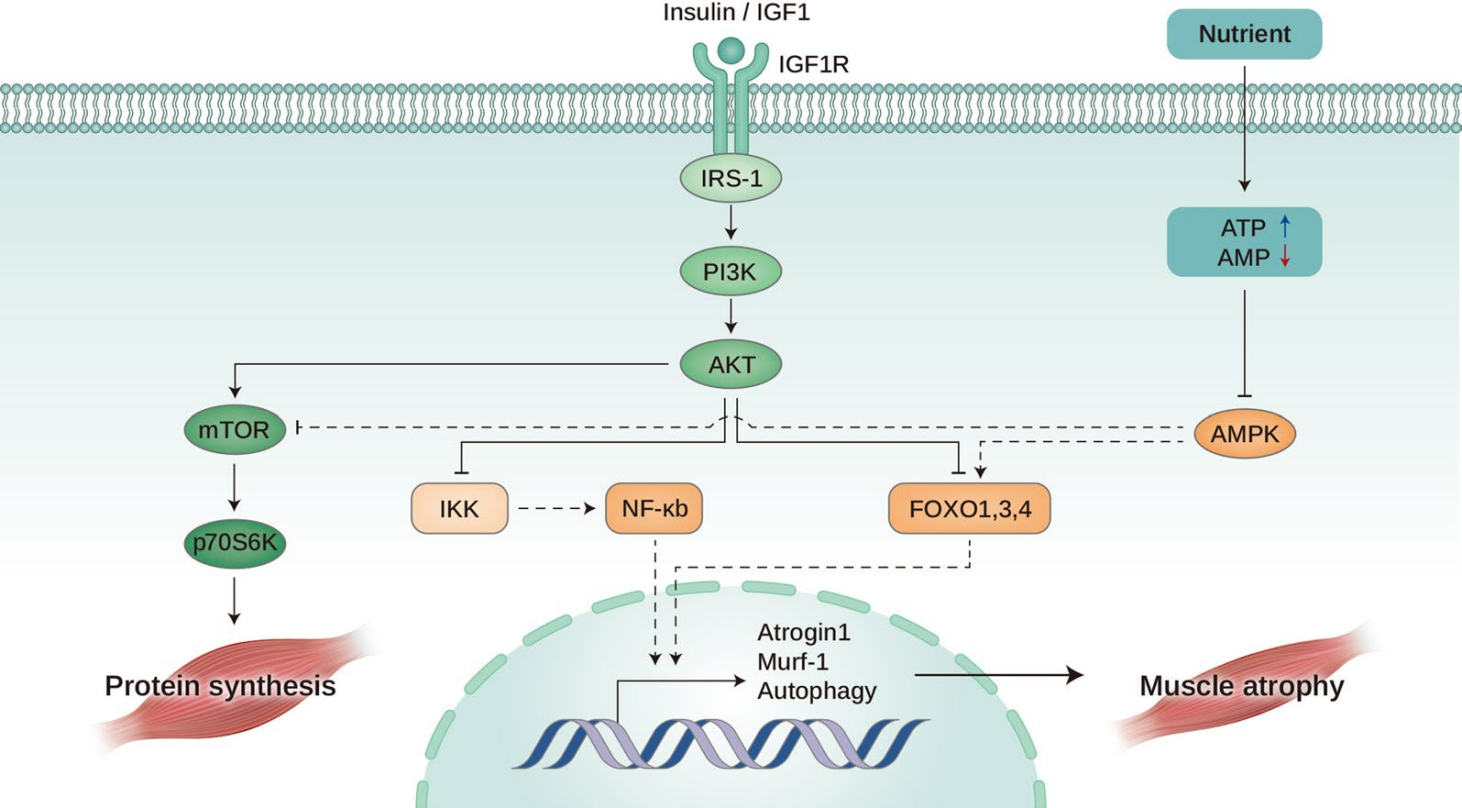
Anabolic pathway leading to muscle growth**.** Insulin or IGF1 binds to the IGF1R and activates IRS-1 which leads to PI3K-AKT-mTOR pathway activation. AKT activates IKK inhibitor and further inhibits the NF-kB pathway, which is implicated in muscle atrophy induction. Furthermore, AKT also negatively regulates the FoxO protein that is responsible for protein degradation. Besides AKT, the mTOR pathway is also activated by nutrients, leading to phosphorylation of S6K that induces protein synthesis and muscle growth. IGF1, Insulin-like growth factor 1; IGF1R, IGF1 receptor; IRS-1, Insulin receptor substrate 1; and OXPHOS, Oxidative phosphorylation. The dashed lines indicate inhibited pathways

Insulin and IGF1 activate a cascade of phosphorylation of key regulators for skeletal muscle growth, differentiation, and homeostasis (Fig. 3) [11, 140]. Therefore, this pathway is essential for protein synthesis and degradation, cellular proliferation and survival, glucose uptake, and energy production [11]. The production of systemic growth factors is regulated by growth hormones. The pancreas produces insulin, and the liver predominantly produces IGF1. Insulin plays a critical role in regulating muscle proteolysis [141–143]. In septic rat, administration of insulin alleviated the degradation of skeletal muscle protein by inhibiting ubiquitin proteasome system [141], while insulin-resistant diabetes db/db mice exhibited accelerated degradation of skeletal muscle protein through the activation of ubiquitin–proteasome pathway [141, 144]. Additionally, decreased whole-body protein synthesis has been linked to insulin resistance in patients with non-small cell lung cancer [145, 146]. Thus, insulin resistance in cancer cachexia can contribute to muscle wasting. Insulin signaling activates signaling molecules that overlap with the ubiquitin–proteasome pathway, which is involved in muscle wasting. Studies have demonstrated the presence of insulin resistance in both human and animal models of catabolic diseases [142]. In patients with cancer cachexia, insulin resistance has been linked with decreased glucose tolerance and insulin sensitivity, leading to a reduction in glucose uptake [142]. In an insulin-resistant state, there is a reduction in P13K and Akt phosphorylation, which inhibits the release of FoxO and caspase-3, resulting in an increase in proteolytic activity [143]. Therefore, maintaining insulin sensitivity is crucial to prevent muscle wasting in patients with cancer cachexia.

IGF1 is also produced by extrahepatic tissues and plays a predominantly autocrine/paracrine role by acting on the same extrahepatic tissues or nearby cells. IGF1 expression is known to sustain muscle growth and regeneration in mice models [11]. The expression of IGF1 or *igf1* mRNA transcripts promotes myotube differentiation and prevents dexamethasone (DEX)-induced atrophy in mouse myotubes [147]. It has been reported that cancer patients with cachexia exhibit lower levels of circulating IGF1 [36, 148, 149]. In a cisplatin-induced muscle wasting model, IGF1 administration suppressed muscle atrophy through induction of IGF1/PI3K/AKT signaling and suppression of MuRF1 and atrogin-1 [150]. Consistently, AKT activation has been reported to be sufficient to completely rescue muscle mass in cachectic animals [137]. Similarly, plakoglobin, a desmosomal component that binds to the insulin receptor and PI3K subunit p85, has been reported to induce PI3K-AKT-FoxO signaling [11]. P13K-AKT-FoxO signaling is the central pathway that controls growth and metabolism in all cell types [151]. A recent study also showed that mir204 and miR-33a can target IGF1 and inhibit its expression, which leads to the inhibition of proliferation, migration, and differentiation of mouse myotube cells in vitro [152, 153].

### Bone morphogenetic protein (BMP)/Smad1/5/8

BMP is a TGF-β superfamily cytokine that binds to its receptor to phosphorylate Smad1/5/8. TGF-β family members, such as myostatin and activin A, which respond to Smad2/3, are negative regulators of muscle growth. However, the BMP/Smad1/5 axis has been identified as a positive regulator of muscle mass growth [154, 155]. Sartori et al. reported that BMP reduction in patients and rodent models leads to muscle mass reduction. Cancer-mediated factors, including activin A and IL-6, would trigger BMP inhibitor Noggin expression in the muscle, leading to the inhibition of BMP activity in muscle fibers and motor nerves. This leads to disruption of the neuromuscular junction (NMJ), denervation, and muscle wasting [156]. Furthermore, they demonstrated that restoring BMP signaling in tumor-bearing mice preserved muscle mass and even survival [156]. On the other hand, upregulation of BMP7 expression or BMP receptors in the muscle leads to Smad1/5-independent muscle fiber hypertrophy. Furthermore, BMP7 overexpression with Akt-mTOR activation leads to the activation of Smad1/5/8 signaling and inhibits muscle atrophy [157]. Conversely, inhibition of this axis would lead to muscle wasting [96, 154]. Follistatin (FS), a TGF-β family inhibitor, also mediates hypertrophy of muscle fibers as a ligand antagonist for myostatin [158] by regulating satellite cell proliferation, inhibiting myostatin signaling, stimulating Smad1/5/8 activation, and improving neuromuscular junction transmission [11, 159, 160]. Thus, inhibition of myostatin/activins by follistatin leads to muscle hypertrophy.

## Alterations of catabolic pathways in cancer cachexia

**Fig. 4:**
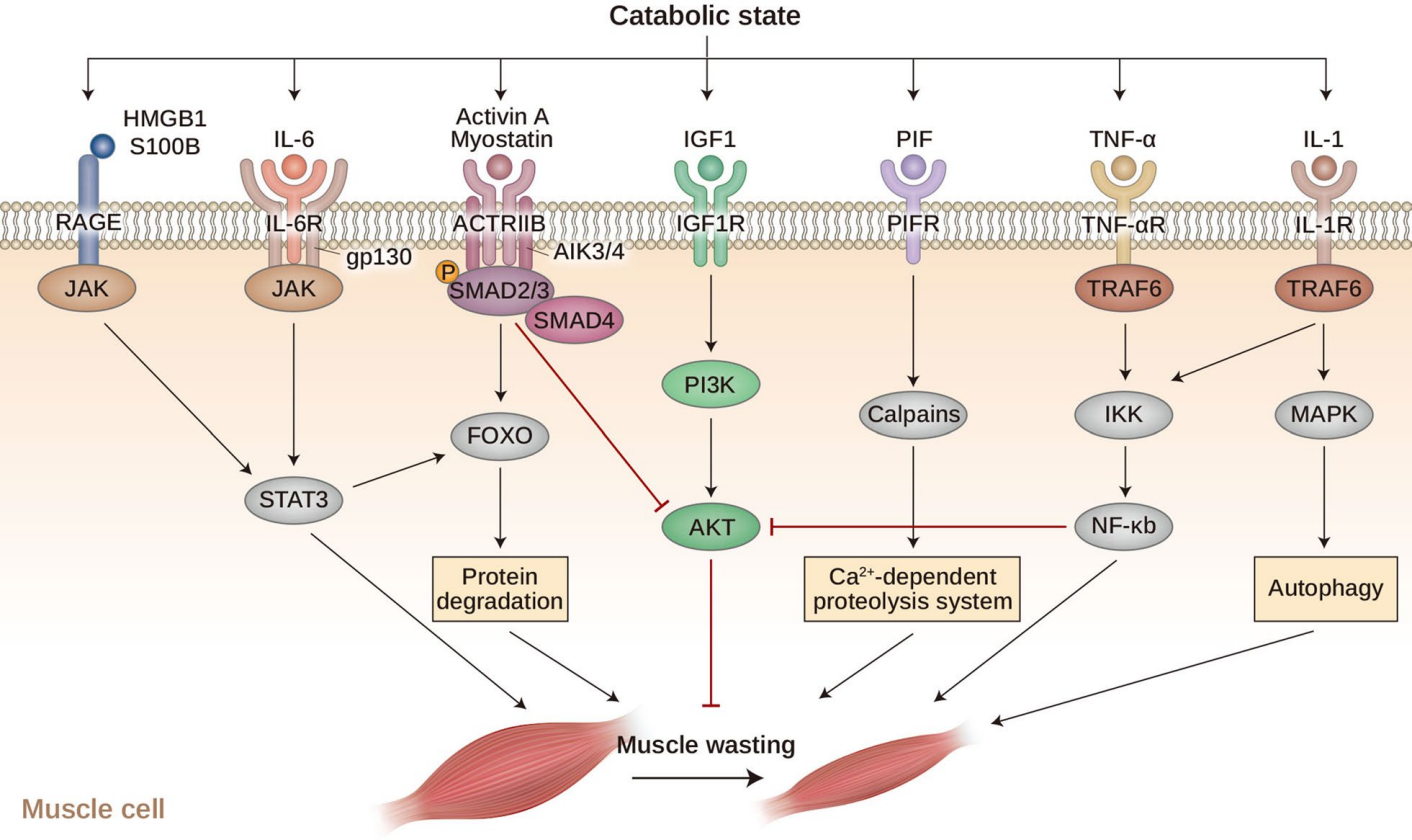
Catabolic pathways lead to muscle atrophy**.** During catabolic states, multiple intracellular signaling pathways are activated and stimulate muscle wasting via protein degradation, Ca2 + -dependent proteolysis system, and autophagy. These catabolic effects in muscle are mediated by specific transcription factors, such as FOXO proteins, NF-κB, and SMAD2 or SMAD3. The activation of these transcription factors results from extracellular stimuli or from stimulation of JAK-STAT signaling and a decrease in the PI3K-AKT-mTOR pathway. Together, these pathways accelerate protein degradation, proteolysis, and autophagy, leading to muscle atrophy. RAGE, receptor for advanced glycation end-product; HMGB1, high mobility group box 1; ActRIIb, activin type II receptor; IGF1, insulin-like growth factor 1; IGF1R, IGF1 receptor; PIF, proteolysis-inducing factor; PIFR, proteolysis-inducing factor receptor; FOXO, forkhead box protein O; and NF-kB, nuclear factor-κB

Skeletal muscle maintenance depends on the balance between dynamic catabolic and anabolic reactions that determine muscle protein levels [161]. The catabolic pathway in skeletal muscle induces muscle loss because of the loss of proteins, organelles, and cytoplasm, which causes cell shrinkage and muscle atrophy [13]. During cancer cachexia, the skeletal muscle undergoes a reduction in protein synthesis and an increase in protein degradation/proteolysis. These changes are associated with organelle dysfunction marked by the upregulation of inflammatory mediator genes, abnormal expression of angiotensin II (AngII), IGF1, and various receptors, proteins, and kinases [121, 162–172]. Changes in skeletal muscle proteins eventually lead to muscle atrophy during cancer cachexia development. Here, we describe several signaling pathways involved in the catabolic pathway of skeletal muscle proteins (Fig. 4).

### Myostatin/activin A

Myostatin is another important pathway that leads to muscle atrophy in certain cachexia models. Myostatin, a member of the TGF-β family, is secreted by muscle cells and circulates in the blood [173–175]. Myostatin is a negative regulator of muscle growth that represses the Akt/mTOR pathway and decreases the number of satellite cells [174]. Myostatin and activin A share the same receptor, activin type 2 receptor B (ActR2B) [176]. A previous study demonstrated that dominant-negative ActR2B in transgenic mice resulted in skeletal muscle hypertrophy [177]. The circulating level of Activin A has been shown to be secreted by cancer cells and elevated in patients with cachexia [175, 178]. In addition, transgenic mice lacking myostatin exhibit increased skeletal muscle mass [179]. Myostatin/activin A upregulate FOXO expression, leading to protein breakdown via MuRF1 and MAFbx/Atrogin1 expression, while they inhibit protein synthesis by repressing the Akt/mTOR signaling pathway via SMAD3 activation [173, 177, 180, 181]. In skeletal muscle, the binding of myostatin/activin A to ActR2B induces the activation of SMAD2 and SMAD3 transcription factors, leading to atrogin-1 expression [182]. Interestingly, myostatin levels were also highly induced by the activation of inflammatory signaling [183, 184]. A cross-sectional study of patients with lung and colorectal cancer demonstrated that high levels of circulating myostatin are correlated with the presence of cachexia syndrome [178]. Although inhibiting myostatin/activin A raises interest in the development of drugs to prevent muscle wasting-related cachexia, the roles of myostatin/activin A in cancer cachexia still need further investigation. This is because the levels of myostatin/activin A often result in opposite effects on muscle atrophy and remain contradictory [178, 185–187].

### NF-κB pathway

NF-κB activation was also identified as a key event in the development of muscle atrophy [188]. In response to TNF-α signaling, NF-κB has been implicated in muscle wasting, which results in the induction of muscle cell death and specific transcriptional regulation inhibiting the IGF1 anabolic pathway [189, 190]. In cancer cachexia, NF-κB also suppresses the expression of MyoD, a muscle-regulatory factor, at the transcriptional level after the activation of TNF-α [191, 192]. In addition to the TNF-α pathway, NF-κB has also been shown to increase MuRF1 expression by activating proteolysis of skeletal muscle proteins [193]. Numerous studies have reported that NF-κB also promotes skeletal muscle atrophy through the iNOS/NO pathway [191, 194]. In primary human skeletal muscle myotubes, the overexpression of NF-κB-inducing kinase (NIK) increased the levels of atrophy markers. In contrast, NIK knockdown resulted in the attenuation of glucocorticoid-induced NIK and Atrogin-1 [193]. More importantly, in a clinical study, NF-κB was found to be highly expressed in patients with cancer cachexia and advanced NSCLC compared to that in healthy patients [168].

### TNFα pathway

Skeletal muscle appears to be significantly vulnerable to cachectic factors, such as inflammatory cytokines [133, 163, 189]. The activation of the UPS is commonly accompanied by the persistent activation of inflammatory mediators, such as TNFα, IL-1β, IL-6, interferon-gamma (IFNγ), and aberrant expression of some essential molecules involved in the inflammatory signaling pathway [133, 163, 170, 189]. TNF-α is an inflammatory factor secreted by macrophages and tumor cells and is also reported to be essential for cachexia-induced muscle atrophy [195, 196]. TNF-α has been reported to directly affect skeletal muscle catabolism by inducing the expression of ubiquitin genes in the UPS [189, 197]. TNF-α exposure has been shown to induce atrogin-1 expression in C2C12 myotubes, while regularly promoting the activation of the p38 MAPK pathway [164]. Additionally, the TNF-α signal was involved in part by the induction of NF-κB, which was then involved in the ubiquitin conjugation and proteasomal degradation of iKb [189]. Therefore, TNF-α may be involved in the UPS of skeletal muscle proteins, leading to muscle atrophy directly and indirectly.

### IL-6-JAK-STAT3 signaling

STAT3 signaling is known to play pivotal roles in multiple types of muscle cells, including skeletal muscle stem cells, myofibers, and macrophages [198]. In skeletal muscle stem cells, STAT3 regulates stem cell function by inhibiting self-renewal [198]. STAT3 promotes myogenic lineage progression in muscle stem cells in an in vivo model by stimulating mitochondrial respiration [199, 200]. In addition, it regulates skeletal muscle mass in myofibers [198, 200]. Increased production of cytokines, such as IFNγ, TNF-α, and IL-6, is a common feature of muscle wasting and cachexia [201]. STAT3 prominently affects muscle wasting and is specifically relevant to IL-6/Janus Kinase (JAK) signaling [202]. The IL-6/JAK/STAT3 signaling has been found to have an important role in cachexia progression by regulating the inflammatory response [162, 203]. The binding of IL-6 to its receptor induces STAT3 phosphorylation, which leads to skeletal muscle proteolysis and muscle wasting [162, 204, 205]. IL-6-mediated IL3 activation has been observed in cachectic patients with gastric and breast cancer [206]. STAT3 also induces apoptosis and muscle atrophy by activating the IκB kinase (IKK)/NF‑κB signaling pathway [194]. STAT3 activation induced a rapid NF‑κB translocation into the nucleus, leading to the binding of NF‑κB to the nitric oxide synthase (iNOS) promoter to activate the iNOS/nitric oxide (NO) pathway that induced the muscle atrophy [194]. STAT3 phosphorylation also increases myostatin, MAFbx, and MURF1 expression. Its phosphorylation stimulates CCAAT/enhancer‑binding protein δ (C/EBPδ) expression and activity, leading to increased expression of myostatin, MAFbx, and MURF1 [207, 208]. Increased levels of STAT3 phosphorylation, C/EBPδ, and myostatin were observed in the Lewis lung carcinoma (LLC) tumor-induced cachexia mouse model [170]. STAT3 also has been reported to be associated with ferroptosis of patient-derived adipose and muscle tissues [209].

Considering the important roles of STAT3 signaling in promoting muscle wasting and cachexia, inhibition of this pathway provides an alternative strategy for the treatment of patients with cachectic cancer. Blocking JAK/STAT3 signaling inhibited skeletal muscle wasting in an IL-6-induced cachexia model [162]. Inhibition of STAT3 activation suppressed caspase-3 and proteolysis, leading to an increase in muscle mass in cancer cachexia [208]. Genetic ablation of STAT3 in a mouse model showed partial amelioration of muscle loss under the induction of diabetes, CDK, and cachexia [207, 208]. Similarly, pharmacological inhibition of STAT3 ameliorates muscle wasting in several mouse models of cancer cachexia [170, 207, 210, 211]. Inhibition of STAT3 signaling using the eukaryotic initiation factor 4A (eIF4A) inhibitor pateamine A alleviated muscle wasting via the translational modulation of inducible iNOS mRNA [201]. STAT3 inhibition using a STAT3 inhibitor (C188-9), JAK2 inhibitor (AG490), sunitinib, and sorafenib (tyrosine kinase inhibitor) partially rescued skeletal muscle loss [170, 210, 211]. As STAT3 inhibition resulted in the amelioration of muscle wasting in animal models, the utility of STAT3 inhibitors provides a promising approach for the treatment of muscle wasting-associated disease. Thus, the therapeutic potential of STAT3 inhibitors deserves to be tested in clinical trials of cachexia-related patients, and the identification of relevant signaling pathways related to STAT3 activation and downstream targets remains to be explored in the future.

### Metabolic dysregulation

Patients with cancer cachexia often experience hypermetabolism, which is frequently accompanied by mitochondrial dysfunction in skeletal muscle, leading to muscle wasting[212]. Studies have demonstrated that dysregulation of mitochondrial metabolism plays a critical role in muscle wasting in the context of cancer cachexia [213, 214]. In preclinical models of cancer cachexia, alterations in mitochondrial dynamics, quality, and function can cause muscle atrophy [213]. Mitochondrial dysfunction, such as increased mitochondrial surface, impairment of mitochondrial dynamics (including increased fission [Fis1], decreased fusion [Mfn1 and Mfn2], or biogenesis [PGC1α]), respiratory chain complexes reduction, and induction of UCP2 and UCP3 gene expression, has been associated with muscle loss [213]. Mitochondrial dysfunction is also linked with the induction of FOXO1/3 by catabolic stimuli [215].

In the skeletal muscle of breast cancer patients, dysregulation of canonical pathways that regulate oxidative phosphorylation and mitochondrial dysfunction has been observed. Additionally, PPAR signaling, which regulates energy metabolism, is reduced and leads to mitochondrial dysfunction through a reduction in β-oxidation. In Lewis lung carcinoma (LLC) mice, tumor progression is negatively correlated with mitochondrial ATP synthesis and induced mitochondrial ROS production [216]. Studies have also shown that in colon cancer patient, there is a reduction in the expression of pyruvate dehydrogenase (PDH), which is essential for mitochondrial energy production. PDH plays a critical role in regulating the entry of carbohydrates into the tricarboxylic acid cycle (TCA cycle), and a reduction in PDH activity can lead to impaired ATP production [217]. In the skeletal muscle of patients with gastrointestinal cancer-associated cachexia, Catro et al*.* found disrupted mitochondrial morphology [218]. Additionally, in older patients with gastric cancer, muscle loss is associated with a reduction in mitochondrial protein content and an increase in mitophagy [167].

## Treatment strategies and clinical trials

**Fig. 5:**
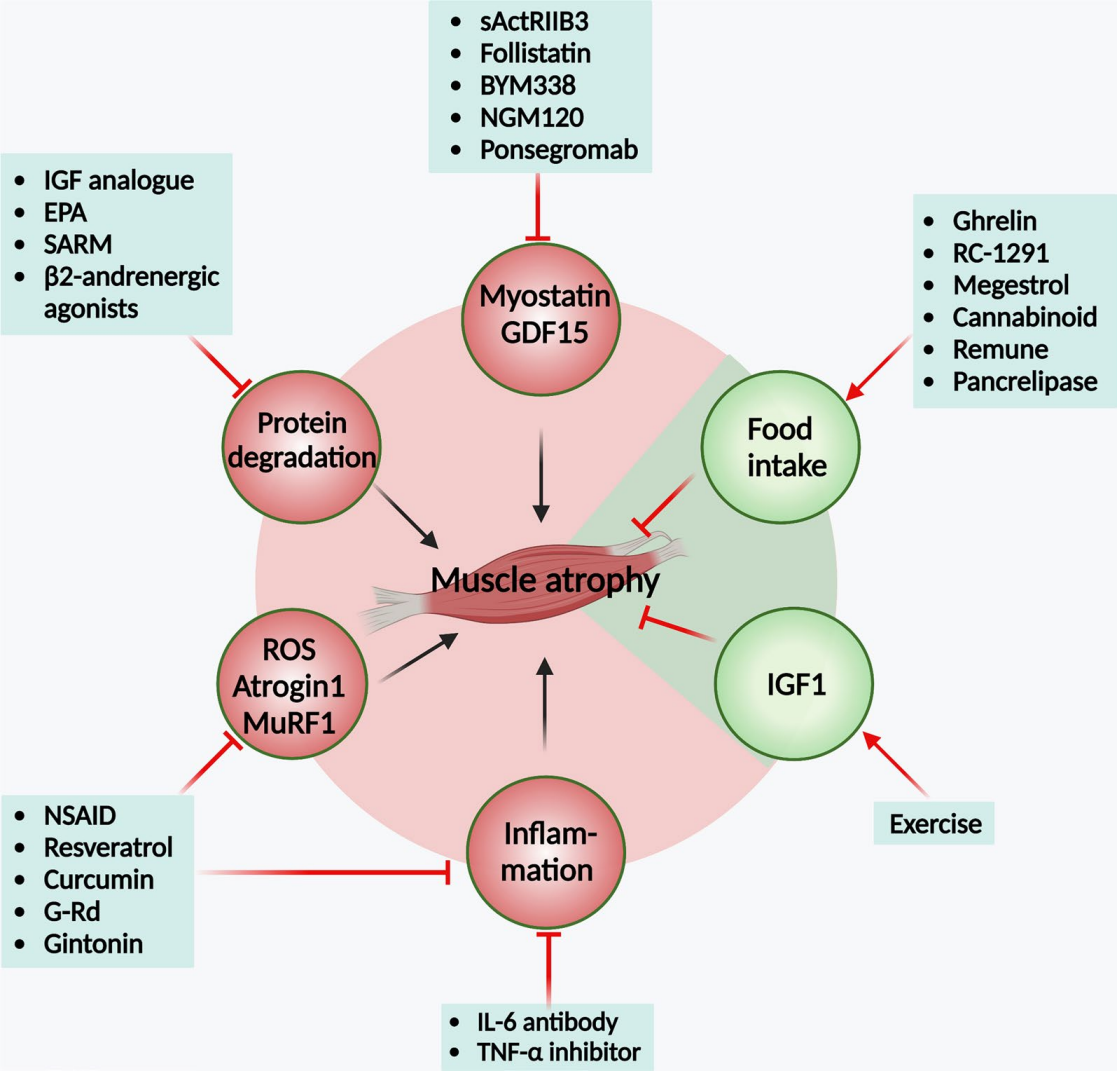
Treatment strategies for cancer cachexia-associated muscle atrophy**.** Several inhibitors are tested to inhibit muscle atrophy caused by protein degradation, ROS, UPS, inflammation, myostatin, and GDF15. On the other hand, exercise, nutrition, and appetite stimulants are used to induce food intake and IGF1, which leads to the inhibition of muscle wasting. TNF-a, tumor necrosis factor-α; IL-6, interleukin 6; ROS, reactive oxygen species; G-Rd, ginsenoside Rd; and IGF1, insulin-like growth fact. The dashed lines indicate inhibited pathways

Because the complexity of the pathogenesis of cancer cachexia-associated muscle atrophy is not completely understood and refractory cachexia is difficult to treat, early diagnosis and intervention are necessary [219, 220]. Multiple factors are involved in cancer cachexia, including anorexia, skeletal muscle wasting, as well as metabolic changes within the body [221–223]. Thus, a comprehensive treatment should be adopted to prevent muscle atrophy caused by cancer. Cancer cachexia treatment should involve not only pharmacological therapy but also multiple interventions, such as nutritional treatment, exercise, and psychosocial interventions [221–224]. The use of promising agents in clinical trials is ongoing and is expected to soon be on the market. In this section, we discuss several treatments that have been conducted as an approach to delineate cancer cachexia (Fig. 5) as well as its application in clinical trials as summarized in Table 2.

**Table 2:** Clinical trials of drugs to ameliorate cancer cachexia. Data from www.clinicaltrials.gov

Compound/drug	Target/agent	Phase	NCT number	Location	Status	Treatment outcomes
Megestrol acetate	Appetite stimulant	III	NCT00002067	USA	Completed	Maximal weight gain was normally achieved within 8 weeks. Unfortunately, the weight gain was mainly due to an increase in fat mass and partly due to edema. No significant effects were reported as regards the Karnofsky index
		II	NCT00002300	USA	Completed	
Ghrelin/Anamorelin HCl	Appetite stimulant	II	NCT01505764	USA	Terminated	Terminated due to poor recruitment (10 subjects were consented, 9 received drug or placebo, 5 completed the study)
		I/II	NCT00933361	Switzerland	Completed	No grade 3/4 toxicity or stimulation of tumor growth was observed. Ghrelin is well tolerated and safe in patients with advanced cancer. No difference was observed between the lower- and upper-dose group for safety, tolerance, and patients’ preference for treatment
		III	NCT01387282	USA	Completed	No differences in grade 3–4 treatment-related adverse events between study groups; the most common grade 3–4 adverse event was hyperglycemia
		III	NCT01387269	USA	Completed	
RC-1291	Ghrelin receptor agonist	II	NCT00267358	USA	Completed	74 patients were eligible for the efficacy analyses. Lean body mass increased in 38 patients in the anamorelin group compared with 36 patients in the placebo group after 12 weeks of the treatment. 42 patients (95%) treated with anamorelin and 33 patients (87%) treated with placebo had adverse events. The most common grade 3–4 adverse events (treatment-related or not) in the anamorelin group were fatigue, asthenia, atrial fibrillation, and dyspnea; in the placebo group, such events were pneumonia and anemia, thrombocytopenia, abdominal pain, anxiety, and dyspnea
		II	NCT00378131	USA	Completed	Not available
Sun11031	Synthetic Ghrelin	II	NCT00698828	Japan	Completed	Not available
BYM338	Myostatin	II	NCT01669174	USA	Completed	BYM338 treatment safely increased skeletal muscle mass but did not improve functional capacity in patients with COPD and low muscle mass. Thigh muscle volume increased at week 4 and remained increased at week 24 in BYM338-treated patients, whereas no changes were observed with placebo. Adverse events in the BYM338 group included muscle-related symptoms, diarrhea, and acne, most of which were mild in severity
Eicosapentaenoic Acid	EPA	Not applicable	NCT00815685	USA	Completed	Not available
NGM120	GDF15 receptor GFRAIL	I	NCT03392116	Australia	Completed	Not available
PF-06946860 (Ponsegromab)	GDF15	I	NCT04299048	USA	Active, not recruiting	Not available
GSK2881078 (SARM)	Androgen receptor	II	NCT03359473	USA	Completed	GSK2881078 was well tolerated, and short-term treatment increased leg strength, when expressed as percent predicted, in men with COPD more than the physical training alone
APD209	Androgen metabolism	II	NCT00895726	UK	Completed	Not available
MT0-102	ß-adrenergic	II	NCT01238107	India	Completed	Not available
VT-122	ß-adrenergic	II	NCT00527319	India	Completed	Not available
ALD518	IL-6	II	NCT00866970	UK	Completed	Not available
Ruxolitinib	JAK/STAT	Early phase I	NCT04906746	USA	Not yet recruiting	Not available
		II	NCT02072057	Switzerland	Terminated	Not available
Curcumin	NF-kB	II	NCT04208334	Thailand	Completed	Not available
Insulatard, flexpen	Insulin	IV	NCT00329615	Sweden	Completed	The total diet energy density did not predict energy balance. Survival was positively, and systemic inflammation negatively associated with energy balance. Only energy intake remained a significant predictor of energy balance after adjustment for survival and inflammatory status
PPP011/CAUM	Cannabis	III	NCT04001010	Canada	Suspended	Not available
Kanglaite	Natural compound		NCT03631459	Beijing	Unknown	Not available
N-acetylcysteine	antioxidant	II	NCT00196885	Germany	Completed	N-Acetylcysteine treatment strongly enhanced the increase in knee extensor strength and significantly increased the sum of all strength parameters if adjusted for baseline arginine level as a confounding parameter. N-acetylcysteine had no significant effect on growth hormone and IGF1 levels but caused a significant decrease in plasma TNF-alpha
Mirtazapine	Antidepressant	II and III	NCT03254173	Egypt	Completed	Not available
		III	NCT03283488	Brazil	Recruiting	On intention-to-treat analysis at week 4, 4 of 17 patients gained 1 kg or more, 1 patient maintained weight (gain of 400 g) and 2 patients lost weight (800 g and 1.2 kg). 24% and 6% of the patients improved appetite and health-related quality of life, respectively
Remune	Nutritional supplement	I	NCT04131426	USA	Recruiting	Not available
Pancrelipase	Appetite stimulant	II	NCT04098237	USA	Recruiting	Not available
Olanzapine	Antipsychotic, neurotransmitter	III	NCT05243251	Egypt	Recruiting	Not available

### Exercise

Currently, the only recommended behavioral treatment for cancer cachexia is exercise [225]. Exercise is considered beneficial in decreasing protein degradation, which reduces various types of atrophy and improves skeletal muscle function [226, 227]. Continuous training improves muscle strength and lean body mass and attenuates inflammatory markers [228, 229]. Exercise has also been reported to increase insulin sensitivity, protein synthesis rates, and antioxidant enzyme activity [230]. According to the American College of Sports Medicine (ACSM), there are several types of exercise regimen for patients with cancer cachexia, such as aerobic, resistance, and flexibility exercise [231]. Aerobic exercise through treadmill running has been shown to suppress cancer cachexia-induced muscle atrophy in vivo by activating adiponectin signaling [142, 232]. In a randomized controlled trial for resistance training for patients with pancreatic cancer, improvements in elbow and knee flexor/extensor muscles were observed, although there were no significant changes in the patients’ body weight [233]. Flexibility training is also a favorable exercise regimen as it mainly aims to increase muscle length [234]. However, it is important to note that exercise is not recommended for patients with frailty, sarcopenia, or other acute illnesses.

### Nutrition and appetite stimulants

Exercise coupled with nutrition therapy is believed to be more beneficial for patients with cachexia. A diet containing 1.5 g/kg/day of protein that constitutes 15–20% of the total caloric intake is highly recommended to overcome the hypercatabolic state during cachexia [228, 235]. Appetite stimulants, such as steroids, progestational agents, and cannabinoids, are early attempts and well-studied nutrition therapies for cachexia [236, 237]. Megestrol acetate (Megace) is a synthetic progestin that is most widely used to stimulate appetite through NPY in the ventromedial hypothalamus or by reducing the synthesis and release of pro-inflammatory cytokines [238, 239]. Clinical trials using high-dose progestin therapy have shown a significant improvement in appetite and body weight. However, later analysis mentioned that the improved body weight was not due to lean body mass, but due to increased fat mass (NCT03254173, 2018; NCT03283488, 2019) [240]. A study of medroxyprogesterone acetate (MPA), another progestin, also showed a similar result [241, 242]. Even though MPA increases appetite and improves quality of life, it has various side effects, such as venous thromboembolism, hypogonadism, adrenal insufficiency, edema, and increased mortality in older patients [237].

Several neuropeptides that regulate appetite are currently undergoing clinical trials for cancer anorexia/cachexia, one of example is the aforementioned ghrelin [41, 50, 243–248]. A phase II randomized clinical trial using ghrelin for patients with cancer cachexia showed that lean body mass, total body mass, and handgrip strength improved in these patients (NCT01505764, 2012) [249]. Anamorelin is a ghrelin receptor agonist that is used in cancer treatment. It promotes ghrelin secretion through ghrelin receptor activation and increases appetite, resulting in increased weight and muscle mass [47]. In early clinical trials, anamorelin improved skeletal muscle mass and appetite [48, 49]. Furthermore, ghrelin intake stimulates energy intake and improves the body weight of cachexia patients, particularly their lean body mass [247, 248]. Thus, ghrelin may counteract anorexia in cancer patients [247].

### Anti-inflammatory drugs

As an increase in pro-inflammatory cytokines is known to be the hallmark of cancer cachexia, targeting inflammatory cytokines has become an interest in the treatment of cachexia [250]. High levels of TNF-α are believed to play a crucial role in cachexia progression [250]. However, the use of anti-TNF-α therapy for cancer-associated cachexia has shown unsatisfying results [251, 252]. Inhibiting TNF-α using a TNF-α receptor blocker and monoclonal antibody also failed to halt muscle atrophy in patient with cachexia [253]. Thalidomide, an immunomodulatory and anti-inflammatory agent, has been evaluated for cancer cachexia treatment because of its potential to decrease TNF-α production, degrade TNF-α mRNA, and inhibit NF-kB pathway activation [254, 255]. It is also suggested that thalidomide can attenuate the signaling pathway initiated by TNF-α, PIF, or ANGII, and inhibit UPS activation [256]. Thalidomide treatment prevents weight loss in several cancer types and cancer cachexia [257]. Although thalidomide showed encouraging results in preventing weight loss in cachexia, other TNF-α inhibitors such as pentoxifylline and infliximab showed no significant improvement in appetite and body weight in a clinical trial [258, 259].

In addition to anti-TNF-α therapy, anti-IL-6 antibody treatment has also been conducted in phases I and II of non-small cell lung cancer and seems to have beneficial effects on the treatment of anemia and cancer cachexia [260]. The cyclooxygenase 2 (COX-2) inhibitor celecoxib has also been evaluated in clinical trials for cancer cachexia. Treatment with 200–300 mg/day celecoxib resulted in a significant improvement in lean body mass and grip strength [261].

### Stimulation of protein synthesis

Stimulating protein synthesis is an alternative treatment for cancer cachexia to overcome and inhibit robust protein degradation in skeletal muscle wasting and cachexia. Growing evidence indicates that increased production of myostatin and its analog activin A plays a role in the progression of atrophy and cachexia [173–175]. The inhibition of myostatin leads to muscle hypertrophy and hence promotes its potency in preventing muscle loss [173–175]. Several interfering agents have been developed to inhibit myostatin-activin A-SMAD signaling, such as follistatin, soluble forms of activin type IIB (ActRIIB), antibodies inhibiting myostatin and its receptor, and recombinant myostatin propeptide. Small molecules that inhibit STAT3 also reduce myostatin levels [177, 262–265]. Treatment with soluble ActRIIB in an in vivo cancer cachexia model prevented skeletal muscle loss and cardiac atrophy, although the levels of circulating TNF-α, IL-1, and IL-6 remained high [175]. ActRIIB treatment prolonged the lifespan of the tumor-bearing mice. The effects exerted by ActRIIB are considered to be due to its ability to inhibit skeletal muscle protein degradation initiated by FOXO3 [175]. While the inhibition of myostatin-activin A signaling showed significant results for muscle wasting and insulin-resistant disease, the effects on satellite cells remained unclear, and additional trials are required to assess the improvement of muscle function after myostatin-activin A inhibition [266–269]. Clinical trials of ActRIIB in patients with dystrophy have been terminated due to unsatisfactory outcomes (NCT01099761, 2010). The clinical trial of another inhibitor, such as BYM338, which specifically inhibits myostatin and activin A, has been tested in a phase II clinical trial (NCT01925209, 2013), and GDF11 is now being considered in clinical trials [263, 270].

Growth differentiation factor 15 (GDF15), a member of TGF-B superfamily, is known to regulate food intake, energy expenditure, and body weight in response to stress [271]. GDF15 expression is elevated in cancer cachexia and associated with reduction of body weight [272–275]. Interestingly, neutralization of GDF15 using mAb restores muscle function and physical performance in cancer cachexia-induced mice model through increasing calorie intake and altering gene expression related to muscle atrophy, catabolism, inflammation, and function [276]. Currently, a phase II clinical trial of ponsegromab, an anti-GDF15 monoclonal antibody, is being conducted (NCT05546476, 2022).

### Other anti-catabolic agents

Although the mechanism of action is still not well understood, increasing muscle mass with testosterone is a well-known strategy to overcome skeletal muscle loss [277, 278]. While testosterone affects protein synthesis through its binding to the muscle-specific androgen receptor (AR), it is reported that testosterone also induces the activation of the PI3K-Akt-mTOR pathway by inducing the transcription of IGF1 [279–281]. Testosterone likely exhibits clinical potential but is likely to be accompanied by adverse side effects. Subsequently, nonsteroidal selective androgen receptor modulators (SARM) are expected to retain anabolic potency with minimal effects on the androgenic pathway [36, 282]. SARMs have been approved for the treatment of men and women with weight-loss catabolic conditions. It has also been shown to increase lean body mass and weight in patients with cancer, HIV, and COPD-related weight loss [36]. Because of their well-tolerated effects, SARMs (Enobosarm, LGD-4033, and MK-0773) are now being used in phase II and phase III clinical trials for patients with cancer [270, 283–286]. Several SARMs are currently being tested in clinical trials (NCT03359473, 2018; NCT02463032, 2015; and NCT02499497, 2016). β2-adrenergic agonists are potent muscle growth promoters that affect muscle hypertrophy and reduce body fat [287, 288]. The long-acting β2-adrenergic agonist, formoterol, has been approved for the treatment of asthma and pulmonary diseases. It also exhibits a potent protective role in skeletal muscle and the heart through its ability to prevent massive protein degradation [289]. Thus, formoterol has the potential to be used to treat skeletal muscle wasting and cachexia. However, while the study of formoterol and other β2-adrenergic agonists, such as clenbuterol, showed promising outcomes in treating muscle atrophy in rats, their application in human trials remains unsatisfactory [290, 291]. Combined treatment with formoterol and megestrol in cancer patients resulted in a small change in muscle mass with no improvement in muscle strength [292]. Similarly, a trial using another inhibitor, espindolol, resulted in improved lean body mass and grip strength, while no increase in the functional parameters was observed [292].

### Natural compounds

Extracts or compounds from commonly consumed dietary foods have attracted attention for the development of anticancer agents. Owing to their safety and efficiency, natural compounds have also been used as agents to prevent muscle wasting and cachexia-associated cancers [293, 294]. Resveratrol, a stilbenoid naturally found in grapes, blueberries, and peanuts is a well-known anticancer agent [295]. In skeletal muscle, resveratrol has been reported to improve mitochondrial biogenesis and inhibit muscle wasting by activating SIRT1 and PGC1a pathways [295]. Resveratrol has also been shown to activate AKT/mTOR signaling, while suppressing E3 ubiquitin ligases by inducing FOXO phosphorylation [296]. Furthermore, in a cancer cachexia mouse model, oral administration of resveratrol inhibited muscle atrophy by reducing the release of immune cytokines [297]. Several studies have suggested that resveratrol prevents protein degradation induced by angiostensin I and dexamethasone [298–300]. Myricanol, a cyclic diarylheptanoid isolated from Chinese bayberry, has also been shown to exert its potential to inhibit muscle wasting-related diseases [153]. Myricanol has been reported to prevent dexamethasone-induced skeletal muscle wasting, particularly by activating SIRT1 signaling [153]. Diarylheptanoids extracted from curcumin have also been reported to block protein degradation and decrease NF-kB nuclear translocation in sepsis models [301, 302]. In cachexia-induced muscle wasting, curcumin treatment also inhibits muscle loss by attenuating lipopolysaccharide-stimulated atrogin-1 expression [303]. Curcumin has been tested in a phase II clinical trial against head and neck cancers (NCT04208334, 2020). Recently, we reported that gintonin, a ginseng-derived lysophosphatidic acid receptor (LPAR) ligand, and ginsenoside Rd (G-Rd) protected myotubes from muscle wasting [304, 305]. Specifically, in vitro and in vivo studies using a Lewis lung carcinoma cell line (LLC1)-induced cancer cachexia mouse model showed that gintonin exhibits anti-atrophy effects that are dependent on the LPAR/Gα signaling axis [304]. We have also demonstrated that G-Rd protects against muscle wasting caused by cancer and aging by interfering with the Stat3 signaling pathway [305].

## Conclusion and future perspective

Cancer cachexia is a metabolic syndrome associated with malignant tumor progression, involving multiple complex mechanisms that induce skeletal muscle atrophy. Cancer cachexia has recently become a major societal concern. Although the molecular mechanisms inducing cachexia have been extensively studied, therapeutic options remain rare, and no drugs have been approved yet. Several therapeutic agents interfering with essential pathways have been shown to improve muscle mass and body weight; clinical trials of these compounds remain unsatisfactory, and the improvement of the quality of life remains difficult to conclude. In this review, we have summarized the molecular signaling pathways involved in cachexia and the therapeutic efforts implemented in patients with cancer cachexia. There are several key take-home messages to consider. Firstly, identifying sensitive biomarkers for earlier clinical intervention stages of cachexia is crucial to achieving better treatment outcomes at the early stage of the cancer diagnosis. Secondly, cancer cachexia affects diverse tissues and metabolic pathways simultaneously. Thus, further study of multidisciplinary treatment for cancer cachexia is necessary. Lastly, further research is needed to define the development of anti-cachexia agents that not only increase muscle mass but also improve physical functions, thus contributing to both alleviating cancer progression and improving the quality of life of patients. Thus, a multi-faceted approach involving the elucidation of novel agents as well as targets will provide useful indications for the development of drugs that can enhance muscle growth and prevent muscle mass loss, which could have great therapeutic importance in the treatment of cancer cachexia.

## Data Availability

Not applicable.
